# Comments on “Transcutaneous tibial nerve stimulation *versus* parasacral stimulation in the treatment of overactive bladder in elderly people: A triple-blinded randomized controlled trial”

**DOI:** 10.6061/clinics/2020/e2214

**Published:** 2020-11-10

**Authors:** Apoorva Srivastav, Nidhi Sharma, Adarsh Kumar Srivastav

**Affiliations:** I Department of Neurological Physiotherapy, Maharishi Markandeshwar Institute of Physiotherapy and Rehabilitation, Maharishi Markandeshwar (Deemed to be University), Mullana, Ambala, Haryana, India; II University Research Fellow, Maharishi Markandeshwar (Deemed to be University), Mullana, Ambala, Haryana, India

Dear Editor,

We went through a recent article published in volume 75 of your esteemed journal, titled “Transcutaneous tibial nerve stimulation *versus* parasacral stimulation in the treatment of overactive bladder in elderly people: A triple-blinded randomized controlled trial” (1). We found the article very resourceful and well written. The treatment intervention used by the authors can be effective in treating overactive bladder symptoms not only in patients receiving tibial nerve stimulation but also in amputees through stimulation of the parasacral nerve. The study was well executed, and the information shared in the article by the authors is quite specific and objective.

However, there are certain aspects that, if considered, could enhance the specificity and knowledge provided by the article. This study had certain limitations that should have been addressed by the authors.

First, in the statistical analysis section, the authors mentioned that they calculated the sample size from the results of a pilot study, but they neither mentioned the reference nor effect size. Since the sample sizes mentioned by the author were different for the two treatment groups, clarity regarding sample size calculation is needed. If sample size calculation was performed based on the assessment of primary and secondary outcomes of a related pilot study, a short table depicting the results of that pilot study could have been included in the article to specify the calculations.

Unfortunately, there was no way to clearly understand how sample size was obtained in this trial. Hence, we used *post hoc* analysis to derive the power of the study. We performed *post hoc* analysis using the primary outcome — the International Consultation on Incontinence Questionnaire (ICIQ)-Overactive Bladder (ICIQ-OAB) scores obtained before and after the test intervention. G* Power software 3.1.9.4 was used for the analysis. By calculating the effect size as 0.52, keeping the sample size at 25 per group, and using the Wilcoxon-Mann-Whitney test (two groups), the power of the study was calculated to be 0.477 or 47.7%. Thus, the power of the study was too low to demonstrate the efficacy with the sample size chosen.

Second, it is important to consider that every trial should be registered before its implementation (2). The authors stated that their clinical trial registry protocol number was Clinical Trials (ReBeC): RBR-9Q7J7Y; however, this number does not correspond to any registered trials. On searching the Brazilian registry of clinical trials, we found no trial registered under this number. If there is any error in the trial registration number, it must be corrected.

Third, allocation concealment ensures that there is no selection bias pertaining to the trial (3). Hence, the authors should have mentioned how the allocation of the participants (to both groups) was concealed. In addition to the assessor and the physiotherapist, the participants should also have been blinded to the allocation.

Fourth, since the authors only enrolled female participants, scales proven valid and reliable for determining quality of life and overactive bladder symptoms in this specific population could have easily been used. Some of these scales are the Overactive Bladder questionnaire (4), Urogenital Distress Inventory-6, and the Incontinence Impact Questionnaire-7. Such scales would have been more suitable than the ICIQ-OAB and ICIQ-Short Form questionnaires, both of which are essentially derived from the same scale.

All other information was presented accurately and precisely in the article. The article is well written, and the language used is easily understandable. The results reported are complete. The authors also included the effect size of every outcome separately, with pre and post values. They mentioned the interventions given to each patient in an easily reproducible manner. Moreover, the subject of study they highlighted was appreciable; this concept is new, and more studies like this one are needed. The findings of the study would also be helpful in the clinical application of transcutaneous parasacral stimulation over the S2, S3, and S4 regions.

If the issues raised above are considered and addressed, the accuracy of this study would be greatly enhanced. It would also be easier for readers as well as researchers to understand the study.

## References

[1] Jacomo RH, Alves AT, Lucio A, Garcia PA, Lorena DCR, de Sousa JB (2020). Transcutaneous tibial nerve stimulation *versus* parasacral stimulation in the treatment of overactive bladder in elderly people: A triple-blinded randomized controlled trial. Clinics.

[2] Viergever RF, Karam G, Reis A, Ghersi D (2014). The quality of registration of clinical trials: Still a problem. PLoS One.

[3] Ferreira CA, Loureiro CA, Saconato H, Atallah A (2011). Validity of Qualis database as a predictor of evidence hierarchy and risk of bias in randomized controlled trials: a case study in dentistry. Clinics.

[4] Lúcio AC, Perissinoto MC, Natalin RA, Prudente A, Damasceno BP, D’Ancona CA (2011). A comparative study of pelvic floor muscle training in women with multiple sclerosis: Its impact on lower urinary tract symptoms and quality of life. Clinics.

